# Entropy Treatment of Evolution Algebras

**DOI:** 10.3390/e24050595

**Published:** 2022-04-24

**Authors:** Farrukh Mukhamedov, Izzat Qaralleh

**Affiliations:** 1 Department of Mathematical Sciences, College of Science, United Arab Emirates University, Al Ain P.O. Box 15551, United Arab Emirates; 2 Department of Mathematical, Faculty of Science, Tafila Technical University, P.O. Box 179, Tafila 66110, Jordan; izzat_math@ttu.edu.jo

**Keywords:** Markov evolution algebra, S-evolution algebra, isomorphism, entropy

## Abstract

In this paper, by introducing an entropy of Markov evolution algebras, we treat the isomorphism of *S*-evolution algebras. A family of Markov evolution algebras is defined through the Hadamard product of structural matrices of non-negative real *S*-evolution algebras, and their isomorphism is studied by means of their entropy. Furthermore, the isomorphism of *S*-evolution algebras is treated using the concept of relative entropy.

## 1. Introduction

The theory of non-associative algebras is an important branch of abstract algebra. Such kinds of algebras include baric, evolution, Bernstein, train, and stochastic algebras. These types of objects were tied up with the abstract description of biological systems [1,2,3,4].

Let E:=(E,·) be an algebra over a field K, where E is called an *evolution algebra* if it admits a basis $B:=\{e_{1},e_{2},\ldots ,e_{n}\}$ such that

(1) $$\begin{array}{c} e_{i}\cdot e_{j}=\left\{\begin{array}{cc} \sum_{k=1}^{n}a_{ik}e_{k},\; & \mathrm{if}\;i=j,\\ 0,\; & \mathrm{if}\;i\neq j. \end{array}\right. \end{array}$$

The matrix $A=a_{ik}$ is called the *structure matrix* of E relative to *B*. A basis *B* satisfying (2) is called the *natural basis* of E. We say that E is a non-negative evolution algebra if K=R and the structure matrix entries $a_{ik}$ are non-negative.

These kinds of algebras were first considered in [5,6,7] and have been exhaustively studied over the recent years (see [8,9,10,11,12,13,14,15,16] and references therein for a review of some of the main results achieved on this topic [17]). These algebras are related to a wide variety of mathematical subjects, including Markov chains and dynamical systems [18,19]. The relationship between evolution algebras and homogeneous discrete-time Markov chains was settled in [6]. We recall that Markov evolution algebra is a non-negative evolution algebra whose structure matrix *A* has row sums equal to 1.

Tian [6] proposed one of the most fruitful further topics of research: the development of the theory of continuous evolution algebras and their connection to continuous-time Markov processes. He outlined continuous evolution algebras to be evolution algebras using multiplication, with respect to a natural basis $B=\{e_{1},e_{2},\ldots ,e_{n}\}$, such that

(2) $$\begin{array}{c} e_{i}\cdot e_{j}=\left\{\begin{array}{cc} \sum_{k=1}^{n}a_{ik}\left(t\right)e_{k},\; & \mathrm{if}\;i=j,\\ 0,\; & \mathrm{if}\;i\neq j. \end{array}\right. \end{array}$$

for some functions $a_{ik}\left(t\right)$.

Recently, Markov evolution algebras have been strongly connected with group theory, Markov processes, the theory of knots, dynamic systems, and graph theory [11,20,21,22,23,24]. In [25], Markov evolution algebras, whose stricture matrices obey semi-group property, were investigated. This type of study is related to the chain of evolution algebras [26].

On the other hand, recently, in [27], we introduced a new class of evolution algebras called *S-evolution algebras*. These algebras are not nilpotent and naturally extended Lotka—Volterra evolution algebras [18]. It is stressed that directed weighted graphs associated with *S*-evolution algebras have meaning, whereas those connected with Lotka—Volterra algebras do not.

Due to [28], the intersection of information theory and algebraic topology is fertile ground. For example, in [29], it was established that the Shannon entropy defines a derivation of the operad of topological simplices. On the other hand, it is important to construct invariants for evolution algebras which can detect their isomorphism. It turns out that such an invariant can be defined via the Shannon entropy for *S*-evolution algebras. In the present paper, we demonstrate how this entropy allows for the treatment of the isomorphism of *S*-evolution algebras. To be precise, we demonstrate that, if *S*-evolution algebras (symmetric) have different entropies, they are not isomorphic. This result enables the construction of many examples of non-isomorphic evolution algebras. As a result of the primary finding, we propose a non-isomorphic family of Markov evolution algebras. This result sheds new light on the Markov evolution algebras and their isomorphism problems.

Let us briefly describe the structure of this paper. Section 2 contains preliminary definitions of evolution algebra. In Section 3, we define the entropy of the structural matrix of the Markov evolution algebra, and we demonstrate that any isomorphic S-evolution algebra would produce the same Markov evolution algebra. Furthermore, we derive Markov evolution algebra through the Hadamard product of the structural matrix *A* of S-evolution algebra. We show that the entropy of such a matrix will be constant if n=2, whereas the entropy will be decreasing if n≥3. This result allows us to construct a lot of non-isomorphic chains of Markov evolution algebras (see [26]). Finally, in Section 4, the relative entropy is defined, and we prove that such a function is a measure of the ‘distance’, even though it is not a metric space, since the symmetric axiom, in general, is not satisfied. In the case of symmetric evolution algebra, we show that this property is satisfied only in the class of isomorphic algebras.

## 2. Preliminaries

In this section, we recall the definitions of S-evolution algebra and some definitions which are needed throughout the paper. Let E be a real non-negative evolution algebra with structure matrix $A=(a_{ik})$ and natural basis *B*. If $0\leq a_{ik}\leq 1$ and

$$\sum_{k=1}^{\infty }a_{ik}=1,$$

for any i,k, then E is called *Markov evolution algebra*. The name is due to the fact that there is an interesting one-to-one correspondence between E and a discrete time Markov chain ${\left(X_{n}\right)}_{n\geq 0}$ with the stated space $\left\{x_{1},x_{2},\ldots ,x_{n},\ldots \right\}$ and transition probabilities given by ${\left(a_{ik}\right)}_{i,k\geq 1}$, i.e., for i,k∈{1,2,…}:

$$a_{ik}=P\left(X_{n+1}=x_{k}|X_{n}=x_{i}\right),$$

for any n≥0.

For the sake of completeness, we wish to state that a discrete-time Markov chain can be thought of as a sequence of random variables $X_{0},X_{1},X_{2},\ldots ,X_{n},\ldots$ defined in the same probability space, taking values from the same set X, and such that the Markovian property is satisfied, i.e., for any set of values $\{i_{0},\ldots ,i_{n-1},x_{n},x_{k}\}\subset X$, and any n∈N, it holds

$$P\left(X_{n+1}=x_{k}|X_{0}=i_{0},\ldots ,X_{n-1}=i_{n-1},X_{n}=x_{i}\right)=P\left(X_{n+1}=x_{k}|X_{n}=x_{i}\right).$$

Notice that, in the correspondence between the evolution algebra E and the Markov chain ${\left(X_{n}\right)}_{n\geq 0}$, what we have is each state of X identified with a generator of *B*.

**Definition** **1** ([27]). *A matrix $A={\left(a_{ij}\right)}_{i,j=1}^{n}$ is called an S-matrix if*

1 *$a_{ii}=0$ for all 1≤i≤n;*
2 *$a_{ij}\neq 0$ if and only if $a_{ji}\neq 0$.*

We notice that, if $A={\left(a_{ij}\right)}_{i,j=1}^{n}$ is a *S−matrix*, then there is a family of injective functions ${\left\{f_{ij}:K\to K\right\}}_{1\leq i<j\leq n}$, with $f_{ij}\left(0\right)=0$ such that $a_{ji}=f_{ij}\left(a_{ij}\right)$ for all 1≤i<j≤n. Hence, each *S*-matrix is uniquely defined by off diagonal upper triangular matrix ${\left(a_{ij}\right)}_{i<j}$ and a family of functions ${\left(f_{ij}\right)}_{i<j}$. This allows us to construct lots of examples of *S*-matrices.

Given an upper triangular matrix ${\left(a_{ij}\right)}_{i<j}$, one can construct several examples of *S*-matrices as follows:

- symmetric matrices, i.e., $f_{ij}\left(x\right)=x$;
- skew-symmetric matrices, i.e., $f_{ij}\left(x\right)=-x$;
- $f_{ij}\left(x\right)={\left(-1\right)}^{i+j}x$.

**Definition** **2.** *An evolution algebra E is called an S-evolution algebra if its structural matrix is an S-matrix.*

**Remark** **1.** *We note that evolution algebras corresponding to skew-symmetric matrices are called Lotka–Volterra evolution algebras. Such kinds of algebras have been investigated in [18].*

One can see that the conical form of the table of multiplication of S-evolution algebra E with respect to *natural basis*$\left\{e_{1},e_{2},...,e_{n}\right\}$ is given by

(3) $$\begin{array}{ccc} & & e_{i}\cdot e_{j}=0,\;i\neq j; \end{array}$$

(4) $$\begin{array}{ccc} & & e_{i}\cdot e_{i}=\sum_{k=1}^{i-1}f_{ki}\left(a_{ki}\right)e_{k}+\sum_{m=i+1}^{n}a_{im}e_{m}. \end{array}$$

We note that, if i=1, then the first part of (4) is zero, if i=n, then the second part is zero.

**Remark** **2.** *The motivation behind introducing S-evolution algebra is that such algebras have certain applications in the study of electrical circuits, finding the shortest routes and constructing a model for analysis and solution of other problems [8,30].*

**Definition** **3.** *A linear map* $\psi :E_{1}\to E_{2}$ *is called the homomorphism of evolution algebras if* ψ(uv)=ψ(u)ψ(v) *for any* $u,v\in E_{1}.$ *Moreover, if ψ is bijective, then it is called an isomorphism. In this case, the last relationship is denoted by* $E_{1}≅E_{2}.$

**Definition** **4.** *Let E be an evolution algebra with a natural basis $B=\{e_{1},\ldots ,e_{n}\}$ and structural matrix $A=\left(\alpha_{ij}\right)$.*

*1.*  *A graph Γ(E,B)=(V,E), with V={1,…,n} and $E=\{\left(i,j\right)\in V\times V:\alpha_{ij}\neq 0\}$, is called the* graph attached to the evolution algebra E relative to the natural basis *B*.
*2.*  *The triple $\Gamma^{w}\left(E,B\right)=\left(V,E,\omega \right)$, with Γ(E,B)=(V,E) and where ω is the map E→F given by $\omega \left((i,j)\right)=\alpha_{ij}$, is called the* weighted graph attached to the S-evolution algebra E relative to the natural basis *B*.

Recall that if every two vertices of a graph are connected by an edge, then such a graph is called *complete*.

Using the graph Γ(E,B), in [27], we have established the isomorphism of *S*-evolution algebras.

**Theorem** **1** ([27]). *Let $E_{1}$ and $E_{2}$ be two S-evolution algebras with ${\left(a_{ij}\right)}_{i,j=1}^{n},\;{\left(b_{ij}\right)}_{i,j=1}^{n}$ structural matrices, respectively, whose attached graphs are complete. Then, $E_{1}≅E_{2}$ if and only if the following conditions are satisfied*

$$\begin{array}{ccc} {\left(\frac{a_{ij}}{b_{ij}}\right)}^{2}\left(\frac{f_{ij}\left(a_{ij}\right)}{g_{ij}\left(b_{ij}\right)}\right) & = & {\left(\frac{a_{ik}}{b_{ik}}\right)}^{2}\left(\frac{f_{ik}\left(a_{ik}\right)}{g_{ik}\left(b_{ik}\right)}\right),\;1\leq i<j<k\leq n.\\ \left(\frac{a_{pi}}{b_{pi}}\right){\left(\frac{f_{pi}\left(a_{pi}\right)}{g_{pi}\left(b_{pi}\right)}\right)}^{2} & = & {\left(\frac{a_{ik}}{b_{ik}}\right)}^{2}\left(\frac{f_{ik}\left(a_{ik}\right)}{g_{ik}\left(b_{ik}\right)}\right),\;1\leq p<i<k\leq n.\\ \left(\frac{a_{pi}}{b_{pi}}\right){\left(\frac{f_{pi}\left(a_{pi}\right)}{g_{pi}\left(b_{pi}\right)}\right)}^{2} & = & \left(\frac{a_{qi}}{b_{qi}}\right){\left(\frac{f_{qi}\left(a_{qi}\right)}{g_{qi}\left(b_{qi}\right)}\right)}^{2},\;1\leq p\leq q<i\leq n. \end{array}$$

## 3. S-Evolution Algebras and Corresponding Markov Evolution Algebras

In what follows, we always assume that E is a non-negative, symmetric *S*-evolution algebra with structure matrix $A=(a_{ik})$ and natural basis *B*. Using the matrix *A*, one can define a stochastic matrix $P\left(A\right)=\left(t_{ij}\right)$ as follows:

(5) $$t_{ij}=\frac{a_{ij}}{\sum_{m=1}^{n}a_{im}},$$

where i,j∈{1,⋯,n}. Sometimes, $t_{ij}$ is denoted by $P(a_{ij})$.

An evolution algebra with the natural basis *B* and structural matrix P(A) is a *Markov evolution algebra* corresponding to E which is denoted by $\bar{E}$.

Our task now is to examine the isomorphism between E and $\bar{E}.$

**Theorem** **2.** *Let (E,A) be a non-negative symmetric S-evolution algebra whose attached graphs are complete, and let $\left(\bar{E},P\left(A\right)\right)$ be its corresponding Markov evolution algebra. Then, $E≅\bar{E}$ if the following conditions are satisfied.*

*1.* 
$\sum_{m=1}^{n}a_{jm}=\sum_{m=1}^{n}a_{km},\;1<j<k\leq n,$

*2.* 
$\sum_{m=1}^{n}a_{pm}=\sum_{m=1}^{n}a_{km},\;1\leq p<k-1\leq n,$

**Proof.**  We notice that E and $\bar{E}$ are S−evolution algebras. So, the isomorphism between these two algebras can be checked by Theorem 1. Hence, the proof is straightforward. □

Consider a discrete random variable X with possible values $\left\{x_{1},x_{2},\ldots ,x_{n}\right\}$ and probability mass function P(X). The entropy can be explicitly written as:

(6) $$\begin{array}{c} H\left(X\right)=-\sum_{i=1}^{n}p\left(x_{i}\right)\ln\left(p\left(x_{i}\right)\right), \end{array}$$

where it is assumed that $0\ln\left(0\right)=\lim_{p\to 0^{+}}p\ln\left(p\right)=0$.

Now, given a non-negative symmetric S-evolution algebra E with structure matrix $\left(a_{ij}\right)$, we define its entropy as follows:

(7) $$\begin{array}{c} H\left(A\right)=-\sum_{i,j=1}^{n}t_{i,j}\ln\left(t_{i,j}\right), \end{array}$$

where $\left(t_{ij}\right)$ is defined by (5).

**Remark** **3.** *We notice that the considered entropy has a relationship with the Jamiolkowski entropy of a stochastic matrix [31]. Indeed, given a stochastic matrix $P={\left(P_{ij}\right)}_{i,j=1}^{n},$ one associates a probability distribution as follows:*

$$D_{P}=\frac{1}{n}\left(P_{11},\ldots ,P_{1n},P_{21},\ldots ,P_{2n},\ldots ,P_{n1},\ldots ,P_{nn}\right).$$

*The Jamiolkowski entropy of P is defined by $h\left(P\right):=H\left(D_{P}\right).$ One can see*

(8) $$\begin{array}{ccc} h(P) & = & -\sum_{i,j}\frac{P_{ij}}{n}\ln\left(\frac{P_{ij}}{n}\right) \end{array}$$

(9) $$\begin{array}{ccc} & = & -\frac{1}{n}\sum_{i,j}P_{ij}\ln\left(p_{ij}\right)+\frac{1}{n}\sum_{i,j}P_{ij}\ln\left(n\right) \end{array}$$

(10) $$\begin{array}{ccc} & = & -\frac{1}{n}\sum_{i,j}P_{ij}\ln\left(p_{ij}\right)+1. \end{array}$$

*Hence, the entropy given by (7) can be represented as follows:*

(11) $$H\left(A\right)=n\left(h(P(A))-1\right).$$

*The obtained formula *(11)* allows us to investigate H(A) in terms of h(P(A)), which has certain applications in information theory. Moreover, all properties of the Shannon entropy can be applied to H(A).*

On the other hand, if one defines the entropy of a stochastic matrix in the sense of [32], then, via *(11)*, one can introduce other types of entropy of evolution algebras. Moreover, given an evolution algebra E with the structure matrix A with $\left(A^{*}A=1\right)$, we may define a mapping $\Phi :C^{n}\to C^{n}$ by $\Phi \left(x\right)=A^{*}xA,$ which defines a quantum channel. Using its Jamiolkowski entropy, we define the entropy of E as follows:

$$H\left(E\right)=n\left(h(\Phi )-1\right).$$

*This will allow us to further investigate the algebraic structure of E with relation to the quantum channel *Φ* [33].*

**Theorem** **3.** *Let $E_{1}≅E_{2}$ be the non-negative symmetric S- evolution algebras with structural matrices $A={\left(a_{ij}\right)}_{1\leq i,j\leq n}$ and $B={\left(b_{ij}\right)}_{1\leq i,j\leq n},$ respectively. Assume that their attached graphs are complete. Then, the corresponding Markov evolution algebras are the same.*

**Proof.**  Let $E_{1}≅E_{2}$. Due to the isomorphism between theses two algebras, we have

(12) $$\begin{array}{c} \frac{a_{ij}}{a_{lm}}=\frac{b_{ij}}{b_{lm}},\;1\leq i\neq j\leq n,\;1\leq l\neq m\leq n. \end{array}$$

The corresponding Markov evolution algebras have the following matrices of structural constants:

$$P\left(A\right)={\left(\frac{a_{ij}}{\sum_{k=1}^{n}a_{ik}}\right)}_{1\leq i,j\leq n},\;P\left(B\right)={\left(\frac{b_{ij}}{\sum_{k=1}^{n}a_{ik}}\right)}_{1\leq i,j\leq n},$$

respectively. Let $a_{i_{0}j_{0}}$ be an arbitrary entry of the matrix A, then

$$P\left(a_{i_{0}j_{0}}\right)=\frac{a_{i_{0}j_{0}}}{\sum_{k=1}^{n}a_{i_{0}k}}.$$

We may assume that $i_{0}\neq j_{0}$ (since the matrices are *S*-matrices). Hence,

$$P\left(a_{i_{0}j_{0}}\right)=\frac{a_{i_{0}j_{0}}}{\sum_{k=1}^{n}a_{i_{0}k}}=\frac{a_{i_{0}j_{0}}}{a_{i_{0}j_{0}}\sum_{k=1}^{n}\frac{a_{i_{0}k}}{a_{i_{0}j_{0}}}}=\frac{1}{\sum_{k=1}^{n}\frac{a_{i_{0}k}}{a_{i_{0}j_{0}}}}.$$

From (12), one has

$$P\left(a_{i_{0}j_{0}}\right)=\frac{1}{\sum_{k=1}^{n}\frac{a_{i_{0}k}}{a_{i_{0}j_{0}}}}=\frac{1}{\sum_{k=1}^{n}\frac{b_{i_{0}k}}{b_{i_{0}j_{0}}}}=\frac{b_{i_{0}j_{0}}}{b_{i_{0}j_{0}}\sum_{k=1}^{n}\frac{b_{i_{0}k}}{b_{i_{0}j_{0}}}}=\frac{b_{i_{0}j_{0}}}{\sum_{k=1}^{n}b_{i_{0}k}}=P\left(b_{i_{0}j_{0}}\right).$$

Hence,

$$P\left(a_{i_{0}j_{0}}\right)=P\left(b_{i_{0}j_{0}}\right).$$

Due to the arbitrariness of $i_{0},j_{0},$ we obtain P(A)=P(B). This completes the proof. □

**Corollary** **1.** *Assume that all conditions of Theorem 3 are satisfied. Then H(A)=H(B).*

**Remark** **4.** *We stress that the converse of Corollary 1 need not be true. Indeed, let $E_{1}$ and $E_{2}$ be two non-negative S-evolution algebras with the following matrices of structural matrices:*

$$\begin{array}{c} A_{1}=\left(\begin{array}{ccc} 0 & a & b\\ a & 0 & c\\ b & c & 0 \end{array}\right) \end{array},\;A_{2}=\left(\begin{array}{ccc} 0 & b & a\\ b & 0 & c\\ a & c & 0 \end{array}\right)$$

*Using the condition from *(12)*, we have $E_{1}≇E_{2}$ for any a≠b. However, $H\left(A_{1}\right)=H\left(A_{2}\right).$*

**Remark** **5.** *The advantage of Theorem 3 is that, for any two non-negative S-evolution algebras whose matrix of structural constants is symmetric, if their entropies are different, then theses algebras are not isomorphic.*

The natural question that arises is: if we have arbitrary isomorphic evolution algebras, are their entropies equal? The following example gives a negative answer.

**Example** **1.** *Let $E_{1}$ and $E_{2}$ be two dimensional evolution algebras with structural matrices*

$$A_{1}=\left(\begin{array}{cc} 0 & 1\\ 1 & 1 \end{array}\right),\;A_{2}=\left(\begin{array}{cc} 0 & 16\\ 2 & 4 \end{array}\right).$$

*Clearly, $E_{1}≅E_{2}.$ Now,*

$$P\left(A_{1}\right)=\left(\begin{array}{cc} 0 & 1\\ \frac{1}{2} & \frac{1}{2} \end{array}\right),\;P\left(A_{2}\right)=\left(\begin{array}{cc} 0 & 1\\ \frac{1}{3} & \frac{2}{3} \end{array}\right).$$

*Now, we may calculate the entropies for both, which are $H\left(A_{1}\right)=\ln\left(2\right)$, $H\left(A_{2}\right)=\ln\left(3\right)-\frac{2}{3}\ln\left(2\right).$ Thus $H\left(A_{1}\right)\neq H\left(A_{2}\right).$*

Given the S-evolution algebra (E,A), the Hadamard product is defined as $M_{t}:a_{ij}A\to \underset{t-\mathrm{times}}{\underset{︸}{A⊙A⊙\ldots ⊙A}}$

(13) $$M_{t}\left(a_{ij}\right)=a_{ij}^{t},\;\;t>0$$

Let us denote $\underset{t-\mathrm{times}}{\underset{︸}{A⊙A⊙\ldots ⊙A}}$ by $A^{{⊙}^{t}}.$ By $\left(E,A^{{⊙}^{t}}\right)$ we denote the evolution algebra whose structural matrix is $A^{{⊙}^{t}}$.

**Lemma** **1.** *Assume that all conditions of Theorem 3 are satisfied and $dim(E_{1})\geq 3$. Then*

*1.*  *If $\frac{a_{ij}}{a_{lm}}=\frac{b_{ij}}{b_{lm}}=1,\;\mathrm{for}\;\mathrm{all}\;\;1\leq i\neq j\leq n,\;1\leq l\neq m\leq n.$ Then $\left(E_{1},A^{{⊙}^{t_{1}}}\right)≅\left(E_{2},B^{{⊙}^{t_{2}}}\right)$ for any $t_{1},t_{2}.$*
*2.*  *$\frac{a_{ij}}{a_{lm}}=\frac{b_{ij}}{b_{lm}}\neq 1,\;\mathrm{for}\;\mathrm{some}\;\;1\leq i\neq j\leq n,\;1\leq l\neq m\leq n.$ Then $\left(E_{1},A^{{⊙}^{t_{1}}}\right)≅\left(E_{2},B^{{⊙}^{t_{2}}}\right)$, if and only if $t_{1}=t_{2}.$*

**Proof.**  Let $A={\left(a_{ij}\right)}_{i,j=1}^{n}$ and $B={\left(b_{ij}\right)}_{i,j=1}^{n}$ be the structure matrices of $E_{1}$ and $E_{2}$, respectively, then $\left(E_{1},A_{1}\right)≅\left(E_{2},A_{2}\right)$, if and only if

$$\frac{a_{ij}}{a_{lm}}=\frac{b_{ij}}{b_{lm}},\;1\leq i<j\leq n,\;1\leq l<m\leq n.$$

Assume that

$$\frac{a_{ij}}{a_{lm}}=\frac{b_{ij}}{b_{lm}}=1,\;\mathrm{for}\;\mathrm{all}\;\;1\leq i<j\leq n,\;1\leq l<m\leq n,$$

then

$${\left(\frac{a_{ij}}{a_{lm}}\right)}^{t_{1}}={\left(\frac{b_{ij}}{b_{lm}}\right)}^{t_{2}}=1.$$

for any $t_{1},\;t_{2}.$ Next, let $\frac{a_{ij}}{a_{lm}}=\frac{b_{ij}}{b_{lm}}\neq 1,\;\mathrm{for}\;\mathrm{some}\;\;1\leq i\neq j\leq n,\;1\leq l\neq m\leq n.$ Then

$${\left(\frac{a_{ij}}{a_{lm}}\right)}^{t_{1}}={\left(\frac{b_{ij}}{b_{lm}}\right)}^{t_{2}}$$

if and only if $t_{1}=t_{2}.$ □

**Remark** **6.** *From above lemma, we emphasize the following points:*

*1.*  *If $dim(E_{1})=2,$ then $\left(E_{1},A^{{⊙}^{t_{1}}}\right)$ and $\left(E_{2},B^{{⊙}^{t_{2}}}\right)$ are isomorphic for any $t_{1},\;t_{2}.$*
*2.*  *If $dim(E_{1})\geq 3$ with $\frac{a_{ij}}{a_{lm}}=\frac{b_{ij}}{b_{lm}}\neq 1,\;\mathrm{for}\;\mathrm{some}\;\;1\leq i\neq j\leq n,\;1\leq l\neq m\leq n,$ and if $t_{1}\neq t_{2}$, then $\left(E_{1},A^{{⊙}^{t_{1}}}\right)$ and $\left(E_{2},B^{{⊙}^{t_{2}}}\right)$ are not isomorphic.*

Now, let us consider $P(A^{{⊙}^{t}})$, which is defined by

$$P\left(A^{{⊙}^{t}}\right)=\left(\frac{a_{ij}^{t}}{\sum_{k=1}^{n}a_{ik}^{t}}\right).$$

Let us denote

$$f_{i}\left(t\right)=\ln\left(\frac{x_{i}^{t}}{\sum_{k=1}^{n}x_{k}^{t}}\right).$$

Then one has

(14) $$\begin{array}{ccc} f_{i}^{\prime }\left(t\right) & = & \frac{\sum_{k\neq i}^{n}x_{k}^{t}\ln\left(\frac{x_{i}}{x_{k}}\right)}{\sum_{k=1}^{n}x_{k}^{t}} \end{array}$$

(15) $$\begin{array}{ccc} f_{i}^{\prime \prime }\left(t\right) & = & -\frac{\sum_{k\neq m}^{n}x_{k}^{t}x_{m}^{t}{\left(\ln\left(\frac{x_{k}}{x_{m}}\right)\right)}^{2}}{{\left(\sum_{l=1}^{n}x_{l}^{t}\right)}^{2}}. \end{array}$$

Let

$$y:=\sum_{j=1}^{n}f_{j}\left(t\right)=\sum_{j=1}^{n}\ln\left(\frac{a_{1j}^{t}}{\sum_{j=1}^{n}a_{1j}^{t}}\right).$$

**Theorem** **4.** *The entropy of *(13)* is related to the following first-order differential linear equation:*

$$ty^{\prime }-y=H\left(A^{{⊙}^{t}}\right).$$

**Proof.**  Let us calculate the entropy of (13). Due to the symmetry of $A^{{⊙}^{t}}$, it is enough to find the value $H(A^{{⊙}^{t}})$ for the first row, the rest will process in the same manner. Hence, the value of $H(A^{{⊙}^{t}})$ at *i* will be denoted by $H(A^{{⊙}^{t,[i]}})$, where

$$H\left(A^{{⊙}^{t}}\right)=\sum_{i=1}^{n}H\left(A^{{⊙}^{t,[i]}}\right)$$

$$\begin{array}{ccc} -H(A^{{⊙}^{t,[1]}}) & = & \frac{a_{12}^{t}}{\sum_{j=1}^{n}a_{1j}^{t}}\ln\left(\frac{a_{12}^{t}}{\sum_{j=1}^{n}a_{1j}^{t}}\right)+\frac{a_{13}^{t}}{\sum_{j=1}^{n}a_{1j}^{t}}\ln\left(\frac{a_{13}^{t}}{\sum_{j=1}^{n}a_{1j}^{t}}\right)\\ & + & \ldots +\frac{a_{1n}^{t}}{\sum_{j=1}^{n}a_{1j}^{t}}\ln\left(\frac{a_{1n}^{t}}{\sum_{j=1}^{n}a_{1j}^{t}}\right)\\ & = & \frac{a_{12}^{t}+\left(a_{13}^{t}+\ldots +a_{1n}^{t}\right)-\left(a_{13}^{t}+\ldots +a_{1n}^{t}\right)}{\sum_{j=1}^{n}a_{1j}^{t}}\ln\left(\frac{a_{12}^{t}}{\sum_{j=1}^{n}a_{1j}^{t}}\right)\\ & + & \frac{a_{13}^{t}}{\sum_{j=1}^{n}a_{1j}^{t}}\ln\left(\frac{a_{13}^{t}}{\sum_{j=1}^{n}a_{1j}^{t}}\right)+\ldots +\frac{a_{1n}^{t}}{\sum_{j=1}^{n}a_{1j}^{t}}\ln\left(\frac{a_{1n}^{t}}{\sum_{j=1}^{n}a_{1j}^{t}}\right). \end{array}$$

The last equality can be rewritten as

(16) $$\begin{array}{ccc} -H(A^{{⊙}^{t,[1]}}) & = & \ln\left(\frac{a_{12}^{t}}{\sum_{j=1}^{n}a_{1j}^{t}}\right)+t\left(\frac{a_{13}^{t}}{\sum_{j=1}^{n}a_{1j}^{t}}\ln\left(\frac{a_{13}}{a_{12}}\right)+\ldots +\frac{a_{1n}^{t}}{\sum_{j=1}^{n}a_{1j}^{t}}\ln\left(\frac{a_{1n}}{a_{12}}\right)\right). \end{array}$$

Now, let

$$f_{1}\left(t\right)=\ln\left(\frac{a_{12}^{t}}{\sum_{j=1}^{n}a_{1j}^{t}}\right),$$

then, from (14) we have

$$f_{1}^{\prime }\left(t\right)=\frac{a_{13}^{t}}{\sum_{j=1}^{n}a_{1j}^{t}}\ln\left(\frac{a_{12}}{a_{13}}\right)+\ldots +\frac{a_{1n}^{t}}{\sum_{j=1}^{n}a_{1j}^{t}}\ln\left(\frac{a_{12}}{a_{1n}}\right).$$

Thus, Equation (16) can be rewritten as follows:

(17) $$\begin{array}{c} -H\left(A^{{⊙}^{t,[1]}}\right)=f_{1}\left(t\right)-tf_{1}^{\prime }\left(t\right). \end{array}$$

Therefore,

$$H\left(A^{{⊙}^{t}}\right)=\sum_{i=1}^{n}H\left(A^{{⊙}^{t,[i]}}\right)=t\left(f_{1}^{\prime }\left(t\right)+f_{2}^{\prime }\left(t\right)+\ldots +f_{n}^{\prime }\left(t\right)\right)-\left(f_{1}\left(t\right)+f_{2}\left(t\right)+\ldots +f_{n}\left(t\right)\right).$$

The last expression leads to the required assertion. This completes the proof. □

Let us denote with $K_{i}$ the set of all maximum entries of the row $R_{i}:=\left(a_{i1},a_{i2},\ldots ,a_{in}\right).$ In what follows, we assume that $|K_{i}|=m_{i}$, $1\leq m_{i}\leq n-1.$

**Theorem** **5.** *Let E be a non-negative symmetric S-evolution algebra with the structural matrix $A={\left(a_{ij}\right)}_{1\leq i,j\leq n}$ with attached graphs; it is complete. The following statements hold true:*

*(i)*  *$H(A^{⊙t})$ is non-increasing, when n≥3;*
*(ii)* 
$\lim_{t\to \infty }H\left(A^{⊙t}\right)=\sum_{i=1}^{n}\ln\left(m_{i}\right);$

*(iii)* 
$\sum_{i=1}^{n}\ln\left(m_{i}\right)\leq H\left(A^{⊙t}\right)\leq n\ln\left(n-1\right).$

**Proof.**  (i). From Theorem 4, we infer that $H\left(A^{⊙t}\right)=ty^{\prime }-y$ where $y=\sum_{i=1}^{n}f_{i}\left(t\right)$ and $f_{i}\left(t\right)=\ln\left(\frac{x_{i}^{t}}{\sum_{k=1}^{n}x_{k}^{t}}\right).$ So, the first derivative of $H(A^{⊙t})$ is given by $H^{\prime }\left(A^{⊙t}\right)=ty^{\prime \prime }=t\sum_{i=1}^{n}f_{i}^{\prime \prime }\left(t\right).$ Next, computing the second derivative of $f_{i}\left(t\right)$ we have

$$f_{i}^{\prime \prime }\left(t\right)=-\frac{\sum_{k\neq m,\;k\neq i,\;m\neq i}^{n}{\left(x_{k}x_{m}\right)}^{t}{\left(\ln\left(\frac{x_{k}}{x_{m}}\right)\right)}^{2}}{{\left(\sum_{l=1}^{n}x_{l}^{t}\right)}^{2}}.$$

Clearly, from the last equation, we find $f_{i}^{\prime \prime }\left(t\right)<0,\;1\leq i\leq n$, then $y^{\prime \prime }<0.$ As t>0, then $H^{\prime }\left(A^{⊙t}\right)=ty^{\prime \prime }<0.$ Hence, $H(A^{⊙t})$ is decreasing. This completes the proof of (i).

Now consider (ii). For the sake of simplicity of calculations, we may assume that $P\left(A^{⊙t}\right)={⋃}_{i=1}^{n}A^{\left[i\right]},$ where $A^{\left[i\right]}$ represent the $i^{th}$ row of $P(A^{⊙t}).$

$$\lim_{t\to \infty }H\left(A^{⊙t}\right)=\lim_{t\to \infty }\sum_{i=1}^{n}H\left(A^{\left[i\right]}\right).$$

where

$$H\left(A^{\left[i\right]}\right)=\sum_{k\neq i}^{n}\frac{a_{k}^{t}}{\sum_{j=1}^{n}a_{j}^{t}}\ln\left(\frac{\sum_{j=1}^{n}a_{j}^{t}}{a_{k}^{t}}\right).$$

$$\lim_{t\to \infty }H\left(A^{\left[i\right]}\right)=\lim_{t\to \infty }\sum_{m\in K_{i}}\left[\frac{a_{m}^{t}}{\sum_{j=1}^{n}a_{j}^{t}}\ln\left(\frac{\sum_{j=1}^{n}a_{j}^{t}}{a_{m}^{t}}\right)\right]+\lim_{t\to \infty }\sum_{k\notin K_{i}}\left[\frac{a_{k}^{t}}{\sum_{j=1}^{n}a_{j}^{t}}\ln\left(\frac{\sum_{j=1}^{n}a_{j}^{t}}{a_{k}^{t}}\right)\right].$$

Simple calculations yields that

$$\lim_{t\to \infty }\sum_{k\notin K_{i}}\left[\frac{a_{k}^{t}}{\sum_{j=1}^{n}a_{j}^{t}}\ln\left(\frac{\sum_{j=1}^{n}a_{j}^{t}}{a_{k}^{t}}\right)\right]=0.$$

Since $|K_{i}|=m_{i},$ then

$$\lim_{t\to \infty }\sum_{m\in K_{i}}\left[\frac{a_{m}^{t}}{\sum_{j=1}^{n}a_{j}^{t}}\ln\left(\frac{\sum_{j=1}^{n}a_{j}^{t}}{a_{m}^{t}}\right)\right]=m_{i}\frac{1}{m_{i}}\ln\left(m_{i}\right)=\ln\left(m_{i}\right).$$

Therefore, $\lim_{t\to \infty }H\left(A^{⊙t}\right)=\sum_{i=1}^{n}\ln\left(m_{i}\right).$ This completes proof (ii).

From the (ii), the maximum value of $H(A^{⊙t})$ occurs at t=0. Putting t=0 in the expression

$$H\left(A^{\left[i\right]}\right)=\sum_{k\neq i}^{n}\frac{a_{k}^{t}}{\sum_{j=1}^{n}a_{j}^{t}}\ln\left(\frac{a_{k}^{t}}{\sum_{j=1}^{n}a_{j}^{t}}\right),$$

we get $H\left(A^{\left[i\right]}\right)=\left(n-1\right)\left(\frac{1}{n-1}\ln\left(\frac{1}{n-1}\right)\right)=\ln\left(n-1\right).$ But $H\left(A^{⊙t}\right)={⋃}_{i=1}^{n}H\left(A^{\left[i\right]}\right).$ This implies that the maximum value of $H\left(A^{⊙t}\right)=n\ln\left(n-1\right).$ On the other hand, from (i) and (ii), we obtain that the minimum value of $H\left(A^{⊙t}\right)=\sum_{i=1}^{n}\ln\left(m_{i}\right).$ Hence, $\sum_{i=1}^{n}\ln\left(m_{i}\right)\leq H\left(A^{⊙t}\right)\leq n\ln\left(n-1\right)$ which yields (iii). □

**Corollary** **2.** *If $|K_{i}|=1,$ then the following statements hold true:*

*1.* 
$\lim_{t\to \infty }H\left(A^{⊙t}\right)=0.$

*2.* 
$0\leq H\left(A^{⊙t}\right)\leq n\ln\left(n-1\right).$

**Remark** **7.** *From Theorem 5, we emphasize the following points:*

*1.*  *In Theorem 5 (i), if n=2, then $H(A^{⊙t})$ is constant.*
*2.*  *In Theorem 5 (i), if $a_{k}=a_{m},$ for any k and m, then $H(A^{⊙t})$ is constant. In this setting, the entropy reaches its maximum value. Moreover, all the Markov evolution algebras are the same.*
*3.*  *From Theorem 5 and Corollary 1, if the $H(A^{⊙t})$ is decreasing, then we infer that $\left\{\left(E,P\left(A^{⊙t}\right)\right)\right\}$ is a non-isomorphic family of Markov evolution algebras. This kind of result allows us to investigate further properties of the chain of evolution algebras associated with $\left\{\left(E,P\left(A^{⊙t}\right)\right)\right\}$.*
*4.*  *All non-negative S-evolution algebras with the maximum entropy are isomorphic.*

Let us consider the following examples:

**Example** **2.** *Let $E_{1},\;E_{2}$ and $E_{3}$ be three dimensional S-evolution algebras with the following structure matrices, respectively:*

$$A=\left(\begin{array}{ccc} 0 & 2 & 3\\ 2 & 0 & 5\\ 3 & 5 & 0 \end{array}\right),\;B=\left(\begin{array}{ccc} 0 & 5 & 6\\ 5 & 0 & 7\\ 6 & 7 & 0 \end{array}\right),C=\left(\begin{array}{ccc} 0 & 4 & 6\\ 4 & 0 & 10\\ 6 & 10 & 0 \end{array}\right).$$

*Then, $A^{t⊙},B^{t⊙}$, and $C^{t⊙}$ are, respectively, given by:*

$$A^{t⊙}=\left(\begin{array}{ccc} 0 & 2^{t} & 3^{t}\\ 2^{t} & 0 & 5^{t}\\ 3^{t} & 5^{t} & 0 \end{array}\right),B^{t⊙}=\left(\begin{array}{ccc} 0 & 5^{t} & 6^{t}\\ 5^{t} & 0 & 7^{t}\\ 6^{t} & 7^{t} & 0 \end{array}\right),C^{t⊙}=\left(\begin{array}{ccc} 0 & 4^{t} & 6^{t}\\ 4^{t} & 0 & 10^{t}\\ 6^{t} & 10^{t} & 0 \end{array}\right).$$

*Then, the corresponding Markov evolution algebras have the following structure matrices:*

$$P\left(A^{t⊙}\right)=\left(\begin{array}{ccc} 0 & \frac{2^{t}}{2^{t}+3^{t}} & \frac{3^{t}}{2^{t}+3^{t}}\\ \frac{2^{t}}{2^{t}+5^{t}} & 0 & \frac{5^{t}}{2^{t}+5^{t}}\\ \frac{3^{t}}{3^{t}+5^{t}} & \frac{5^{t}}{3^{t}+5^{t}} & 0 \end{array}\right),P\left(B^{t⊙}\right)=\left(\begin{array}{ccc} 0 & \frac{5^{t}}{6^{t}+5^{t}} & \frac{6^{t}}{6^{t}+5^{t}}\\ \frac{5^{t}}{5^{t}+7^{t}} & 0 & \frac{7^{t}}{5^{t}+7^{t}}\\ \frac{6^{t}}{6^{t}+7^{t}} & \frac{7^{t}}{6^{t}+7^{t}} & 0 \end{array}\right)$$

*One can see that $P\left(A^{t⊙}\right)=P\left(C^{t⊙}\right)$.*

*Then,*

$$\begin{array}{ccc} H(A^{t⊙}) & = & t\left(\frac{2^{t}\log\left(\frac{3}{2}\right)}{2^{t}+3^{t}}+\frac{2^{t}\log\left(\frac{5}{2}\right)}{2^{t}+5^{t}}+\frac{3^{t}\log\left(\frac{5}{3}\right)}{3^{t}+5^{t}}\right)\\ & & +\log\left(\frac{2^{t}+3^{t}}{3^{t}}\right)+\log\left(\frac{2^{t}+5^{t}}{5^{t}}\right)+\log\left(\frac{3^{t}+5^{t}}{5^{t}}\right)\\ H(B^{t⊙}) & = & t\left(\frac{5^{t}\log\left(\frac{6}{5}\right)}{6^{t}+5^{t}}+\frac{5^{t}\log\left(\frac{7}{5}\right)}{5^{t}+7^{t}}+\frac{6^{t}\log\left(\frac{7}{6}\right)}{6^{t}+7^{t}}\right)\\ & & +\log\left(\frac{6^{t}+5^{t}}{6^{t}}\right)+\log\left(\frac{5^{t}+7^{t}}{7^{t}}\right)+\log\left(\frac{6^{t}+7^{t}}{7^{t}}\right) \end{array}$$

**Figure 1:**
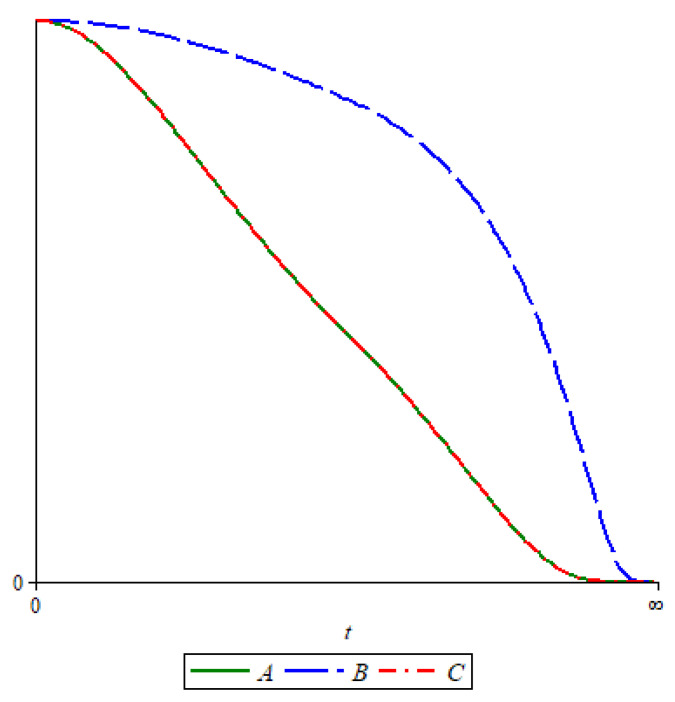
Graph of $H\left(A^{t⊙}\right),H\left(B^{t⊙}\right)$.

*Figure 1 shows the graphs of $H\left(A^{t⊙}\right),H\left(B^{t⊙}\right)$, and $H(C^{t⊙})$, and, since $E_{1}≅E_{3}$ and $E_{2}≇E_{1}$, one can see that the graphs of $H(A^{t⊙})$ and $H(C^{t⊙})$ are identical, whereas the graph of $H(B^{t⊙})$ is different.*

The following example is related to (4) of remark (18).

**Example** **3.** *Let $E_{1},\;E_{2}$ be three dimensional S-evolution algebras with the following structure matrices, respectively:*

$$A=\left(\begin{array}{ccc} 0 & 1 & 1\\ 1 & 0 & 1\\ 1 & 1 & 0 \end{array}\right),\;B=\left(\begin{array}{ccc} 0 & 2 & 2\\ 2 & 0 & 2\\ 2 & 2 & 0 \end{array}\right).$$

*Then, $A^{t⊙}$ and $B^{t⊙}$ are, respectively, given by:*

$$A^{t⊙}=\left(\begin{array}{ccc} 0 & 1 & 1\\ 1 & 0 & 1\\ 1 & 1 & 0 \end{array}\right),B^{t⊙}=\left(\begin{array}{ccc} 0 & 2^{t} & 2^{t}\\ 2^{t} & 0 & 2^{t}\\ 2^{t} & 2^{t} & 0 \end{array}\right).$$

*Then, the corresponding Markov evolution algebras have the following structure matrices:*

$$P\left(A^{t⊙}\right)=\left(\begin{array}{ccc} 0 & \frac{1}{2} & \frac{1}{2}\\ \frac{1}{2} & 0 & \frac{1}{2}\\ \frac{1}{2} & \frac{1}{2} & 0 \end{array}\right),P\left(B^{t⊙}\right)=\left(\begin{array}{ccc} 0 & \frac{1}{2} & \frac{1}{2}\\ \frac{1}{2} & 0 & \frac{1}{2}\\ \frac{1}{2} & \frac{1}{2} & 0 \end{array}\right)$$

*One can see that $P\left(A^{t⊙}\right)=P\left(B^{t⊙}\right)$.*

*Then,*

$$\begin{array}{c} H\left(A^{t⊙}\right)=H\left(B^{t⊙}\right)=3\ln2. \end{array}$$

*Since $P(A^{t⊙})$ and $P(B^{t⊙})$ reach the maximum entropy, $E_{1}≅E_{2}.$*

## 4. Relative Entropy

Suppose that we have two sets of discrete events, $x_{i}$ and $y_{j}$, with the corresponding probability distributions, $\left\{p\left(x_{i}\right)\right\}$ and $\left\{p\left(y_{j}\right)\right\}$. The *relative entropy* between these two distributions is defined by

$$\begin{array}{c} D(p\left(x_{i}\right)\;||\;p\left(y_{i}\right)):=\sum_{i}p\left(x_{i}\right)\ln\left(\frac{p(x_{i})}{p(y_{i})}\right)\;. \end{array}$$

This function is a measure of the ‘distance’ between $\left\{p\left(x_{i}\right)\right\}$ and $\left\{p\left(y_{j}\right)\right\}$, even though it is not metric space, since the symmetric axiom in general is not satisfied D(p(x)||p(y))≠D(p(y)||p(x)).

Let $E_{1}$, $E_{2}$ be non-negative symmetric S-evolution algebras with matrix of structural matrices $A=(a_{ij})$ and $B=(b_{ij})$. Let $P\left(A\right)=\left(t_{ij}\right)$ and $P\left(B\right)=\left(s_{ij}\right)$ be the corresponding stochastic matrices (see (5)). We define the relative entropy of *A* and *B* as follows:

(18) $$\begin{array}{c} D\left(A\;||\;B\right):=\sum_{i,j=1}^{n}t_{i,j}\ln\left(\frac{t_{ij}}{s_{ij}}\right). \end{array}$$

**Theorem** **6.** *Let $E_{1}$, $E_{2}$ be non-negative symmetric S- evolution algebras with structural matrices $A={\left(a_{ij}\right)}_{1\leq i,j\leq n}$ and $B={\left(b_{ij}\right)}_{1\leq i,j\leq n},$ respectively. Assume that their attached graphs are complete. If $E_{1}≅E_{2},$ then $D({A_{1}}^{t⊙}\left|\right|{A_{2}}^{t⊙})=D({A_{2}}^{t⊙}\left|\right|{A_{1}}^{t⊙})=0,$ for any t>0.*

**Proof.**  One can see that

$$P\left({A_{1}}^{t⊙}\right)={\left(\frac{a_{ij}^{t}}{\sum_{j=1}^{n}a_{ij}^{t}}\right)}_{1\leq i,j\leq n},\;\;P\left({A_{2}}^{t⊙}\right)={\left(\frac{b_{ij}^{t}}{\sum_{j=1}^{n}b_{ij}^{t}}\right)}_{1\leq i,j\leq n}.$$

if we write $P({A_{1}}^{t⊙})$ as the row vectors $P\left({A_{1}}^{t⊙}\right)={\left[a^{\left(i\right)}\right]}_{1\leq i\leq n}$ and $P\left({A_{2}}^{t⊙}\right)={\left[b^{\left(i\right)}\right]}_{1\leq i\leq n}$.

Now, we are going to compute $D(a^{\left(i\right)}||b^{\left(i\right)}).$ For a fixed *i*, one has

(19) $$\begin{array}{c} D(a^{\left(i\right)}\left|\right|b^{\left(i\right)})=\sum_{k=1}^{n}\left[\frac{a_{ik}^{t}}{\sum_{j=1}^{n}a_{ij}^{t}}\ln\left({\left(\frac{a_{ik}}{b_{ik}}\right)}^{t}\left(\frac{\sum_{j=1}^{n}b_{ij}^{t}}{\sum_{j=1}^{n}a_{ij}^{t}}\right)\right)\right]. \end{array}$$

If k=n, then

$$\frac{a_{in}^{t}}{\sum_{j=1}^{n}a_{ij}^{t}}=1-\sum_{m=1}^{n-1}\left[\frac{a_{im}^{t}}{\sum_{j=1}^{n}a_{ij}^{t}}\right].$$

Substituting the last expression into (19), we obtain

(20) $$\begin{array}{ccc} D(a^{\left(i\right)}||b^{\left(i\right)})= & & \sum_{k=1}^{n-1}\left[\frac{a_{ik}^{t}}{\sum_{j=1}^{n}a_{ij}^{t}}\ln\left({\left(\frac{a_{ik}}{b_{ik}}\right)}^{t}\left(\frac{\sum_{j=1}^{n}b_{ij}^{t}}{\sum_{j=1}^{n}a_{ij}^{t}}\right)\right)\right]+\\ & & \left(1-\sum_{m=1}^{n-1}\left[\frac{a_{im}^{t}}{\sum_{j=1}^{n}a_{ij}^{t}}\right]\right)\left(\ln\left({\left(\frac{a_{in}}{b_{in}}\right)}^{t}\left(\frac{\sum_{j=1}^{n}b_{ij}^{t}}{\sum_{j=1}^{n}a_{ij}^{t}}\right)\right)\right). \end{array}$$

The last expression can be rewritten as follows:

(21) $$\begin{array}{ccc} D(a^{\left(i\right)}||b^{\left(i\right)})= & & \sum_{k=1}^{n-1}\left[\frac{a_{ik}^{t}}{\sum_{j=1}^{n}a_{ij}^{t}}\ln\left({\left(\frac{a_{ik}}{b_{ik}}\right)}^{t}{\left(\frac{b_{in}}{a_{in}}\right)}^{t}\right)\right]+\\ & & \ln\left({\left(\frac{a_{in}}{b_{in}}\right)}^{t}{\left(\frac{b_{in}}{a_{in}}\right)}^{t}\left(\frac{\sum_{j=1}^{n}\frac{b_{ij}^{t}}{b_{in}^{t}}}{\sum_{j=1}^{n}\frac{a_{ij}^{t}}{a_{in}^{t}}}\right)\right). \end{array}$$

Since $E_{1}≅E_{2},$ we have ${\left(\frac{a_{ik}}{b_{ik}}\right)}^{t}={\left(\frac{b_{in}}{a_{in}}\right)}^{t}$ and $\sum_{j=1}^{n}\frac{b_{ij}^{t}}{b_{in}^{t}}=\sum_{j=1}^{n}\frac{a_{ij}^{t}}{a_{in}^{t}}.$ Hence, from (21), we obtain

(22) $$\begin{array}{c} D(a^{\left(i\right)}\left|\right|b^{\left(i\right)})=\sum_{k=1}^{n-1}\left[\frac{a_{ik}^{t}}{\sum_{j=1}^{n}a_{ij}^{t}}\log\left(1\right)\right]+\log\left(1\right)=0. \end{array}$$

Due to the arbitrariness of i, we arrive at $D(a^{\left(i\right)}||b^{\left(i\right)})=0$ for any *i*. This completes the proof. □

## 5. Conclusions

In this paper, we introduced an entropy of Markov evolution algebras, and treated the isomorphism of the corresponding *S*-evolution algebras. It turns out that the considered entropy is a semi-invariant of non-negative symmetric evolution algebras. This work opens new insight to the isomorphism problem through the entropy theory. Moreover, we have pointed out that entropy can be investigated by means of quantum channels. Furthermore, a family of Markov evolution algebras is defined through the Hadamard product of the structural matrices of non-negative real *S*-evolution algebras, and their isomorphism is studied through entropy. The isomorphism of any algebra is considered a crucial task. So, it is necessary to find a shortcut way that is effective and accurate to study such a problem. This paper treats this problem by using the entropy value in the class of evolution algebras. However, this property is not valid for general evolution algebras, as we have shown in Example 1. Therefore, for other types of algebras, it is better to find other kinds of entropies.

## Data Availability

Not applicable.
